# Phylogenetic footprinting of non-coding RNA: hammerhead ribozyme sequences in a satellite DNA family of *Dolichopoda *cave crickets (Orthoptera, Rhaphidophoridae)

**DOI:** 10.1186/1471-2148-10-3

**Published:** 2010-01-04

**Authors:** Lene Martinsen, Arild Johnsen, Federica Venanzetti, Lutz Bachmann

**Affiliations:** 1 Natural History Museum, Department for Research and Collections, University of Oslo, 0318 Oslo, Norway; 2 Via Giuseppe Berto 31, 00142 Rome, Italy

## Abstract

**Background:**

The great variety in sequence, length, complexity, and abundance of satellite DNA has made it difficult to ascribe any function to this genome component. Recent studies have shown that satellite DNA can be transcribed and be involved in regulation of chromatin structure and gene expression. Some satellite DNAs, such as the *pDo500 *sequence family in *Dolichopoda *cave crickets, have a catalytic hammerhead (HH) ribozyme structure and activity embedded within each repeat.

**Results:**

We assessed the phylogenetic footprints of the HH ribozyme within the *pDo500 *sequences from 38 different populations representing 12 species of *Dolichopoda*. The HH region was significantly more conserved than the non-hammerhead (NHH) region of the *pDo500 *repeat. In addition, stems were more conserved than loops. In stems, several compensatory mutations were detected that maintain base pairing. The core region of the HH ribozyme was affected by very few nucleotide substitutions and the cleavage position was altered only once among 198 sequences. RNA folding of the HH sequences revealed that a potentially active HH ribozyme can be found in most of the *Dolichopoda *populations and species.

**Conclusions:**

The phylogenetic footprints suggest that the HH region of the *pDo500 *sequence family is selected for function in *Dolichopoda *cave crickets. However, the functional role of HH ribozymes in eukaryotic organisms is unclear. The possible functions have been related to *trans *cleavage of an RNA target by a ribonucleoprotein and regulation of gene expression. Whether the HH ribozyme in *Dolichopoda *is involved in similar functions remains to be investigated. Future studies need to demonstrate how the observed nucleotide changes and evolutionary constraint have affected the catalytic efficiency of the hammerhead.

## Background

Noncoding tandem-repetitive satellite DNA (satDNA) has long been known to constitute a large portion of any eukaryotic genome [1]. Recent studies in *Drosophila *through underreplication documented a strong positive correlation between genome size and the amount of satDNA [2]. SatDNA is usually located in the heterochromatic parts of the chromosomes close to the centromeres and telomeres. The high evolutionary turn-over of satDNA and the frequent species-specific patterns have puzzled researchers for many years. Such data challenge the hypothesis that there is a general biological function of satDNA. The enormous diversity of satDNA in nucleotide sequence, length of repeats, complexity, and genomic abundance, instead suggest that a specific function cannot be ascribed to satDNA.

However, transcription of satDNA and other non-coding DNA has been found in many species (e.g. [3-10]). The function of the vast majority of these transcripts is unclear, but recent studies using RNA interference have documented the role of non-coding RNAs in regulation of gene expression, chromatin organization, and genome functioning (for a review, see [11]). For example, it has been demonstrated that small non-coding RNAs can play a crucial role in regulating heterochromatin formation [12] and it has been found that transcripts of tandem repeated non-coding DNA can give rise to dsRNAs [13].

The transcription of satDNA was in some instances also linked to hammerhead (HH) ribozyme activity which may affect certain regulatory mechanisms in the cell. SatDNA-derived HH ribozyme structures have been detected in several organisms as different as schistosomes, cave crickets, and salamanders [14-16]. The HH ribozyme is one of the smallest catalytic RNAs and was first identified in viroid and viroid-like satellite RNA sequences where they catalyze a specific phosphodiester bond isomerization reaction in the course of rolling-circle replication[17,18]. All HH ribozymes detected in animal satDNA so far have been shown to self-cleave in *cis *long multimeric transcripts into monomers [6,14-16,19,20]. The HH ribozyme from *Schistosoma mansoni *is certainly the best characterized of natural HH ribozymes. This particular ribozyme has also been shown to perform efficient ligation [21] and *trans*-cleavage of an RNA target [22]. Recently, an active HH ribozyme was also found in a mammalian messenger RNA that self-cleaved both *in vitro *and *in vivo *and reduced protein expression of C-type lectin type II (*Clec2*) genes in mouse cells [22]. This study showed that ribozymes might also act in post-transcriptional regulation of protein coding gene expression in a way that is similar to destabilizing protein factors.

HH ribozymes are characterized by a conserved central core which is mandatory for cleavage activity. The consensus sequence for a HH ribozyme consists of three paired stems that branch from the core as defined by conserved nucleotides (reviewed in [23]). In addition, the natural HH ribozymes require additional sequence elements outside of the conserved catalytic core to facilitate intracellular activity. These elements are variable and are therefore not phylogenetically conserved [24]. The correlation between structure and function of the HH ribozyme became clear only recently [25-27]. For many years studies of HH ribozymes were restricted to a minimal construct. However, there have been major discrepancies between the crystal structure of this particular construct and the results of biochemical experiments [26,28,29]. The biochemical data could only be explained if a large-scale rearrangement of the core region was assumed during catalysis [28,30,31]. Recently, the HH ribozymes found in schistosomes were characterized in more detail and loop-loop interactions were shown to have a large impact on HH ribozyme activity [25,26,32].

Previously, database searches identified a potential HH ribozyme (49 base pairs) in *Dolichopoda *cave crickets embedded within the *pDo500*satDNA family and self-cleavage of satDNA transcripts has been shown *in vitro *for RNA from *D. baccettii *[15]. The roughly 500 bp repeats of the *pDo500 *have been found in all *Dolichopoda *species studied so far [33] and constitute approximately 5% of the *D. schiavazzii *genome [34]. Phylogenetic analyses of the *pDo500 *sequences from 12 *Dolichopoda *species showed that this satDNA family evolved gradually at a rate of 3.4% per one million year [33]. Thus, *pDo500*-based phylogenetic hypotheses were mainly congruent with the mitochondrial DNA phylogeny [33,35]. The HH ribozyme in *Dolichopoda *performed self-cleavage *in vitro *and this was associated with processing of long multimer transcripts into monomers *in vivo *[15]. For the HH ribozyme of *Dolichopoda *two cleavage mechanisms were suggested: 1) *cis *cleavage by a single-HH (sHH) ribozyme which is the folding of the 49 bp HH sequence, and 2) *trans *cleavage by a double-hammerhead (dHH) which is a hybrid structure between two extended HH sequences of 68 bp.

We took a phylogenetic footprint approach to analyze in detail the *pDo500 *satDNA family from 38 different populations representing 12 species of *Dolichopoda *cave crickets with particular emphasis on the HH region. We assessed whether the HH motif in the *pDo500 *satDNA is more conserved than other parts of the *pDo500 *repeat unit. HH stems should be more conserved than loops, due to their importance for stabilizing secondary structures. Furthermore, the core region was expected invariant. RNA folding patterns of the HH regions of pDo500 sequences from different populations and species may indicate which *Dolichopoda *species are expected to utilize active HH ribozymes. The results indicated that the putative HH motif in the *pDo500 *satDNA is likely to be under selective constraints and may be functional in *Dolichopoda*.

## Results

**Figure 1:**
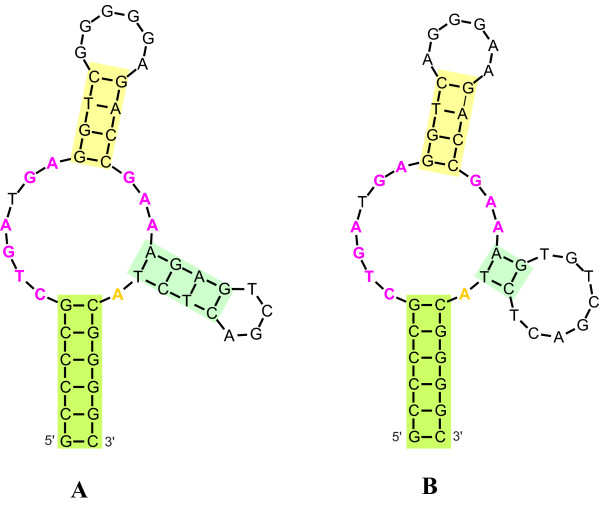
**Folding of two different hammerhead sequences from *Dolichopoda***. Folding of two hammerhead sequences from A) *D. geniculata *(AUS4) and B) *D. schiavazzii *(CPS1). These two structures illustrate the criteria for predicting potentially active HH ribozymes in the populations and species of *Dolichopoda*. A) represents the Pst3 structure and B) represents the For6-2 structure. The figures result from RNAFold analyses. The colors represent: green = stem I, yellow (light) = stem II, blue = stem III, yellow (deep) = cleavage site.

**Figure 2:**
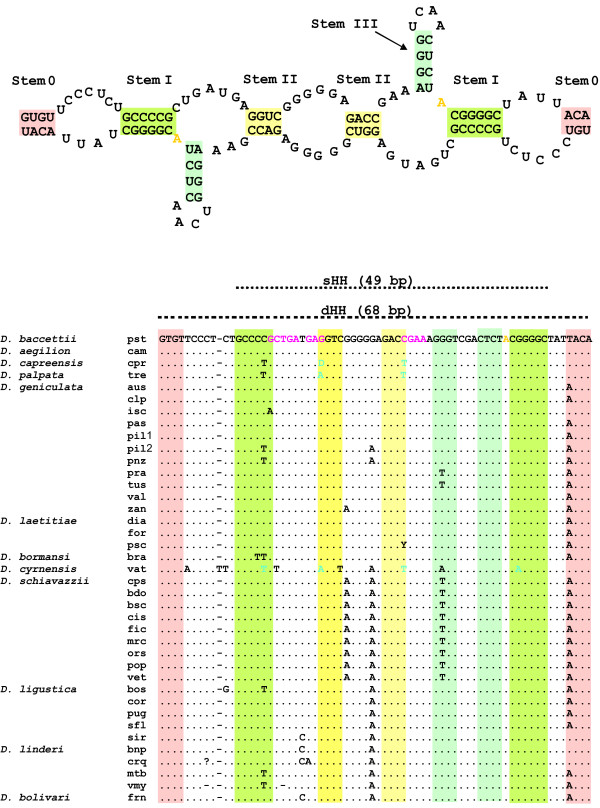
**The dHH structure and the consensus alignment of the sHH (49 bp) and dHH (68 bp) regions from the *pDo500 *satDNA in *Dolichopoda***. The sHH and dHH regions of the *pDo500 *consensus sequences as derived for each population by Martinsen et al., in press [33]. The colors represent: pink = stem 0 green = stem I, yellow (light) = stem II, blue = stem III, yellow (deep) = cleavage site, magenta = invariable core residues, turquoise = compensatory mutations. A dHH structure - as suggested by Rojas et al. (2000) - is made up of two extended sHH sequences (49 bp + 19 bp) that hybridize. Stem I-III are found in both the sHH (see Figure 1) and the dHH structure. For the dHH stem III is folded exactly the same way as in the sHH - i.e. two areas (blue) from the same sequence pair with each other - while pairing of stem I and II requires two different sequences. The figure of the dHH is adopted from Rojas et al. (2000) and edited further to illustrate the alignment of the *pDo500 *consensus sequences. In cpr the D denotes A, T, or G, and in crq the ? denotes C, T, or gap.

We analyzed 198 *pDo500 *satDNA sequences from several populations of 12 *Dolichopoda *species, each represented with 3-9 sequences. The molecular characteristics of these sequences were described in detail elsewhere [33]. The HH region *sensu *Rojas et al. [15] was present in all sequences except in sequence *vat3 *from *D. geniculata *which contains an extended deletion of 308 bp. The *pDo500 *sequences in the data set ranged from 463 bp to 505 bp out of which 49 bp relate to the regular sHH region (Figure 1) and 68 bp to the dHH region. The 68 bp region is a 19 bp extension of the 49 bp region (Figure 2).

We used three different measures in order to assess the level of conservation in the HH and the NHH sections of the *pDo500 *satDNA sequences. Two of these measures - the Shannon-Wiener index and a measure analogous to the homozygosity index - were subjected to a resampling procedure (bootstrap analyses with number of replicates = 1000) and all comparisons yielded significant differences. Both the 49 bp sHH sequence and the 68 bp dHH sequence were significantly more conserved than the NHH section (for a summary, see Table 1).

**Table 1:** Mann-Whitney U Tests of the bootstrapped Shannon-Wiener index and the Homozygosity index (Approach 1 see Methods).

Regions compared	Number of sites compared	Shannon-Wiener index, p-values	Honmozygosity index, p-values
The HH region versus the NHH region	49	p < 0.0001 Z = -6.287	p < 0.0001 Z = -21.664
The stems of the HH region versus the NHH region	28	p < 0.0001 Z = -13.033	p < 0.0001 Z = -19.622
The loops of the HH region versus the NHH region	21	p = 0.019 Z = -2.347	p < 0.0001 Z = -5,542
The stems of the HH region versus the loops of the HH region	21	p < 0.0001 Z = -12.328	p < 0.0001 Z = -12.199
The double HH region versus the NHH region	68	p < 0.0001 Z = -12.328	p < 0.0001 Z =- 28.221
The stems of the double-HH region versus NHH region	35	p < 0.0001 Z =- 30.723	p < 0.0001 Z =- 31,211
The loops of the double-HH region versus the NHH region	32	p < 0.0001 Z =- 5.670	p < 0.0001 Z =- 10.690
The stems of the double-HH region versus the loops of the HH region	32	p < 0.0001 Z =- 29.081	p < 0.0001 Z =- 25.513

For the histograms of the most frequent nucleotide at each position there is a slight trend of values being closer to 1 for the HH region than for the NHH region (see Additional file 1). Nevertheless, the Mann-Whitney U tests showed that this difference is statistically significant only for the 68 bp dHH region (summarized in Table 2). Stems alone were also significantly different from the NHH region, both for the 49 bp and the 68 bp regions. In contrast, loops were not significantly different. According to the most frequent nucleotides found per position, the 49 bp sHH region was not significantly different from the NHH region. However, this is likely to result from the lower sample size of only 49 base pairs for the sHH region compared with the dHH region with 68 bp.

**Table 2:** Mann-Whitney U Tests of the most frequent nucleotide in the HH region versus NHH region (Approach 2 see Methods).

Regions compared	Double-hammerhead (68 bp)	Single-hammerhead (49 bp)
The HH region versus the NHH region	p = 0.001 n1 = 429 n2 = 68 Z = -3.214	p = 0.142 n1 = 448 n2 = 49 Z =- 1.469
The stems of the HH region versus the NHH region	p = 0.006 n1 = 429 n2 = 35 Z = -2.762	p = 0.026 n1 = 28 n2 = 448 Z = -2.226
The loops of the HH region versus the NHH region	p = 0.801 n1 = 429 n2 = 33 Z = -0.252	p = 0.281 n1 = 21 n2 = 448 Z = -1.078
The stems of the HH region versus the loops of the HH region	p = 0.064 N1 = 35 n2 = 33 Z = -1.854	p = 0.457 n1 = 28 n2 = 21 Z = -0.743

Figure 2 illustrates the sequence variation in both the single and the double HH region. The variation affects mainly those bases that are not involved in either the hypothetical base pairing in the stems or the core region in the catalytic centre of the ribozyme. The hypothetical cleavage site is altered in only 2 clones. In the uridine turn most mutations affect position U7, which is the only position in the core region that may vary [26,36]. The most common mutation in U7 is the C→T transition, which is species specific for the two closely related *D. linderi *and *D. bolivari*. The positions C3 and G8 form a Watson-Crick base pair in the three-dimensional structure of the HH ribozyme, and these positions are highly conserved and mutated in only four and two sequences, respectively. G12 which interacts with the cleavage site, is mutated in only one sequence. Of the two other core positions A13 and A14, only A13 is altered in two sequences.

There is a relatively high number of mutations in the stem I region, although the two positions closest to the core [26], i.e. C1.1 and G2.2, are almost invariant. The positions G1.2 and C2.2 are altered in a number of sequences, but frequently these changes are compensatory mutations that sustain the Watson-Crick base pairing. Compensatory mutations were also found in stem II where they affect the conserved positions G10.1 and C11.1. Stem III contains a non-canonical base pair which in most sequences is G-U, which is the most common wobble found in RNA secondary structures [37,38]. The non-canonical base pair U-U was also found in this stem and was species specific to *D. schiavazzii*.

Altogether, there are 19 compensatory mutations distributed in 13 sequences that can potentially restore the base pairing stems (in the total alignment of 198 sequences). Six of them fold into an active HH structure (see below). The remaining seven sequences cannot fold into an active HH structure due to other mutations.

The secondary structure predictions for the 198 *pDo500 *sequences with a HH ribozyme region are summarized in Table 3. The majority of sequences, i.e. 146 out of 198, folded into active HH ribozyme structures according to the criteria specified above. For 33 sequences, the HH ribozyme structure was the first choice, i.e. the structure with the lowest free energy. For the remaining 113 sequences the HH structure was not the structure with the lowest free energy, although the respective values were within the range of those described earlier for the active HH structures of the *pDo500 *sequences *Pst3 *(-20,4) and *For6-2 *(-14,2) [15]. The HH structures that were energetically preferred all showed the *Pst3 *structures, not the HH structures previously described for *For6-2*.

**Table 3:** The number of *pDo500 *sequences per species that contains or lacks the proposed functional HH ribozyme sequence motif.

Taxa	Total number of *pDo500 *sequences per species	Number of sequences with the Pst3-like structure	Number of sequences with the For6-2-like structure	Total number of sequences with a potentially active hammerhead structure (A+B)	Number of sequences without a potentially active hammerhead structure
*D. schiavazzii*	52	3	37	40	12
*D. aegilion*	9	5	0	5	4
*D. linderi*	26	15	0	15	11
*D. bolivari*	5	4	0	4	1
*D. cyrnensis*	2	0	0	0	2
*D. bormansi*	3	0	0	0	3
*D. baccettii*	6	4	0	4	2
*D. laetitia*	12	9	1	10	2
*D. palpata*	4	3	0	3	1
*D. capreensis*	5	3	0	3	2
*D. geniculata*	50	34	10	34	16
*D. ligustica*	20	17	0	17	3

Folding analyses were also performed for population specific consensus sequences as determined previously [33]. Again, the vast majority of sequences, i.e. 34 out of 39, folded into active HH ribozyme structures. The consensus sequences of *D. schiavazzii *and the PRA and TUS populations of *D. geniculata *formed the *For6-2 *structure, whereas all other consensus sequences folded into the *Pst3 *structure.

## Discussion

The HH ribozyme is the best studied ribozyme structure to date [39]. However, HH ribozymes have still not been found in a large number of species. Of the non-viroid organisms, the natural HH ribozyme has primarily been studied in *Schistosoma mansoni*. In the current study, we investigated the conservation of the HH ribozyme sequence, embedded in the *pDo500 *satDNA repeats, in 12 species of *Dolichopoda *cave crickets. This is currently the most comprehensive dataset of HH ribozyme sequences in terms of the number of species involved.

Here we applied a phylogenetic footprinting approach [40,41] in order to assess the phylogenetic conservation of the HH ribozyme region. Using three different measures of sequence variation, we found a significantly higher level of sequence conservation for the HH region than for the NHH region of the *pDo500 *repeats. This clearly suggests an evolutionary constraint on the HH structure in the *pDo500 *satellite family, which has been hypothetized earlier on the basis of self cleavage of *pDo500 *transcripts from *D. baccettii *[15]. In particular pairing stem regions as well as the core of the HH ribozyme are highly conserved in the *pDo500 *satDNA family.

SatDNA is known for its high evolutionary turnover; it may thus be surprising to find highly conserved HH ribozyme sequences within the *pDo500 *satDNA family. However, the *pDo500 *satDNA was characterized earlier as relatively homogeneous. Bachmann et al. [34] noted that the *pDo500 *satDNA was relatively conserved and low in copy number in *D. schiavazzi*, at least compared to the two species-specific satDNA families *pDoP102 *[42] and *pDsPv*400 [34]. Two trends for the mode of evolution of the three satDNA families were detected: 1) a positive correlation of sequence variability and copy number, and 2) a negative correlation between sequence variability and length of repeat. While the first trend was considered in line with the theory on molecular evolution of satDNA, the second trend was not (e.g. [43]. High sequence homogeneity is expected to correlate to high recombination rate, which in turn should reduce repeat length. The results presented here add a further aspect to the mode of evolution of the *pDo500 *satDNA in *Dolichopoda *- the evolutionary constraint brought upon the *pDo500 *repeats by the conserved HH ribozyme may explain the unexpected negative correlation between sequence variability and repeat length described by Bachmann et al. [34].

In the *pDo500 *data set we find compensatory mutations in at least 13 sequences. The compensatory mutations were observed in stems I and II and can restore the hypothetical base pairing in the single-HH structure. If we consider the double-HH, the number of potential compensatory mutations may even be higher because a double-HH consists of two different repeats that need to hybridize. In stem I we found mutations in position 2.2 which in some instances were compensated for by mutations in position 1.2 in the same sequence, but in other instances were not. The compensatory mutations in the stem regions do, to some extent, covary and this is considered further evidence for a functional constraint on *pDo500 *satellite DNA. Although some HH sequences probably have lost the ability to form an active structure, the majority of our single-HH sequences could fold into a potentially active HH structure as previously described [15].

**Figure 3:**
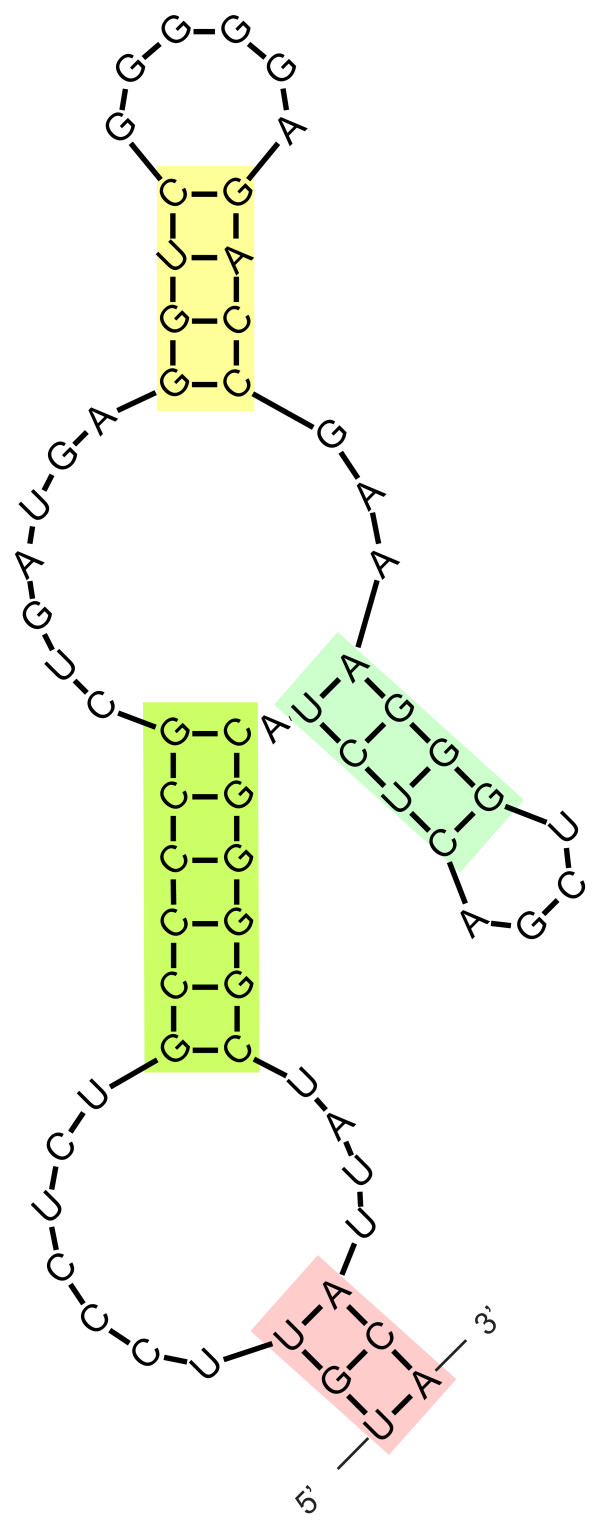
**A putative extended HH structure in *Dolichopoda *cave crickets**. The sequence is the same as for the previously suggested dHH sensu Rojas et al (2000), but here the structure consists of one sequence folding into a HH structure with an extended stem I, as opposed to the dHH that consists of two sequences hybridizing with each other. The color code is as in Figure 1.

The biological role of HH ribozymes that have been uncovered to date can be divided in two groups. One is related to the viroid and viroid-like HH ribozymes which play a role in RNA replication through rolling circle amplification [17,18,44]. The second is the role of the HH ribozymes found in eukaryotic organisms which remains speculative [39]. For example, the newt HH ribozyme, which is embedded in the Satellite 2 family (sat2), has been associated with a ribonucleoprotein (RNP) in the ovaries [45]; an RNA binding protein (NORA) has been characterized as a promising candidate as a constituent of this complex [19]. The function of this complex is unclear; however, it has been associated with *trans *cleavage of an RNA target [45]. The sat2 with its HH ribozyme has been found in nine different species representing four families of amphibians [46]. There are striking similarities between the HH ribozyme in amphibians and *Dolichopoda *as they are both found in satellite DNA transcripts and are conserved between large numbers of species. In addition, the *Dolichopoda *HH also has the potential to form an extended HH structure (Figure 3) as previously described for the amphibian HH ribozyme [46,47]. Whether the HH ribozyme in the *pDo500 *satDNA from *Dolichopoda *is involved in a similar RNP complex as in amphibians needs to be investigated. Neither the well-characterized HH ribozyme from *S. mansoni*, which is also derived from satDNA transcripts, nor the HH ribozyme from *A. thaliana*, have been linked to a function. On the other hand, the split HH ribozyme found in mRNA from rodents has been implicated in regulation of gene expression [22].

## Conclusion

Further functional analyses are certainly necessary to address the biological significance of the *pDo500 *HH ribozyme in *Dolichopoda *cave crickets. Future experiments may focus on the transcription pattern of *pDo500 *sequences in the different *Dolichopoda *species and on the self-cleavage of *pDo500 *repeats that carry nucleotide substitutions in the 49 bp and 68 bp regions described above. However, the data presented herein strongly suggest that the HH ribozyme in *Dolichopoda *cave crickets is an example of sequence conservation in non-coding DNA due to functional importance to the organism. Nevertheless, this is certainly a special case similar to that described earlier in amphibians [19,45]. Coding for functional ribozymes, at present, cannot be considered a general function of satDNA. There are two major known functions related to satDNA: 1) the proper functioning of the centromere [48-51], and 2) processes related to non-coding RNAs. The HH ribozyme in *Dolichopoda *is a special case of category two. The encoding of the HH ribozyme may explain the slow evolutionary rate for the *pDo500 *satDNA family.

## Methods

### Materials

*PDo500 *satDNA sequences from 38 populations representing 12 *Dolichopoda *cave cricket species from Italy and Spain that were previously described were included in the analyses [33,34]. The 198 sequences were downloaded from GenBank; accession numbers GU322143-GU322341 [33]. Details on the cloning and PCR amplification strategies, as well as the sequence alignment of the *pDo500 *satDNA sequences, were described previously [33]. An alignment of the HH region is provided in Additional File 2.

### Sequence Analyses

The region comprising the potential HH structure was compared with the detailed descriptions of recently described active HH ribozymes [22,26,27,36,52]. Numbering of specific nucleotides comprising the HH ribozyme was according to Hertel et al. [53].

Two mechanisms of cleavage were suggested in a previous study of the HH ribozymes in *Dolichopoda *[15]: 1) a single *cis*-cleavage mechanism carried out by a 49 bp sequence folding into a regular HH ribozyme, and 2) a *trans*-cleavage mechanism carried out by a dHH structure where two copies of an extended HH sequence of 68 bp pair with each other. The latter was thought to explain the efficient processing of long primary transcripts of the *pDo500 *satDNA in vivo. The current study focused on both the 49 bp sequence (Figure 1) and the extended 68 bp region (Figure 2).

Genetic variation in the potential HH region was tested against the genetic variation in the remainder of the *pDo500 *sequences following two different approaches: 1) Two different measures of variation were estimated for each position in the alignment and subsequently used in bootstrap approaches. The Shannon entropy [54] was calculated as , where p is the proportion of each character state in each position and S is the number of characters. For calculating the Shannon entropy, gaps were treated as a fifth character state; i.e. S = 4 at positions without gaps and S = 5 at positions with gaps. A variability estimate was calculated in analogy to the homozygosity index [55] as follows H = [A]^ 2^+[T]^ 2 ^[C]^ 2^+[G]^2^. In positions where gaps amount to more than 3% the equation was extended to H = [A]^ 2^+[T]^ 2 ^[C]^ 2^+[G]+[-]^2^. One thousand bootstrap replicates were run for both the Shannon entropy and the homozygosity index. 2) The frequency of the nucleotide at each position of the alignment was determined for both the HH region and the non-HH (NHH) region of the *pDo500 *sequence and illustrated by histograms (see Additional File 1). For all three statistical procedures (Shannon entropy, homozygosity index and tests of nucleotide frequency), each of the following comparisons were made: the HH region versus the NHH region of the *pDo500 *repeats; the stems of the HH region versus the NHH region of the *pDo500 *monomer; the loops of the HH region versus the NHH region of the *pDo500 *monomer; the stems of the HH region versus the loops of the HH region. Each comparison was tested in a Mann-Whitney U test using the program SPSS [56]. Both approaches were used on both the 49 bp and the 68 bp HH regions.

Secondary structure of 198 HH sequences was predicted and illustrated with the programs MFOLD - using the web based program Quickfold that allows folding of many sequences simultaneously on the DINAMelt server [57,58] - and RNAdraw. The proportion of sequences per species that may fold into potentially active HH ribozyme structures was determined. As criteria indicating potentially active HH ribozyme structures, we used secondary structure similarity of the sequences to the structure of either the *Pst3 *or *For6-2 *sequences of the *pDo500 *satDNA family which were previously identified as active HH ribozyme sequences in *Dolichopoda *[15]. In addition, we used invariance of the core region since it was shown that mutations in the core region abolish or significantly reduce HH cleavage [59]. These analyses were done only on the 49 bp HH sequence.

## Authors' contributions

LB and FV planned the project, conducted labwork, and participated in writing and editing the manuscript. LM conducted the labwork, analyzed the data and participated in writing and editing the manuscript. AJ participated in data analyses and writing and editing of the manuscript. All authors approved the final version of the manuscript.

## Supplementary Material

Additional file 1 **Histograms of the most frequent nucleotide at each position in the *pDo500 *alignment**. Histograms of the most frequent nucleotide at each position in the alignment. See Materials and Methods for details. Click her for the file

Additional file 2 **Alignment of the HH sequences of all *pDo500 *sequences from *Dolichopoda***. The alignment of all the 198 HH sequences from *Dolichopoda*. Color codes are as in Figure 2. Click her for the file
