# Supplementary material for: Phylogenetic footprinting of non-coding RNA: hammerhead ribozyme sequences in a satellite DNA family of Dolichopoda cave crickets (Orthoptera, Rhaphidophoridae)
Source: BMC Evol Biol. 2010 Jan 4;10:3. doi: 10.1186/1471-2148-10-3 (PMC2837043; doi:10.1186/1471-2148-10-3)
Supplement: Additional file 2 — Alignment of the HH sequences of all pDo500 sequences from Dolichopoda. The alignment of all the 198 HH sequences from Dolichopoda. Color codes are as in Figure 2. [file 1471-2148-10-3-S2.pdf]

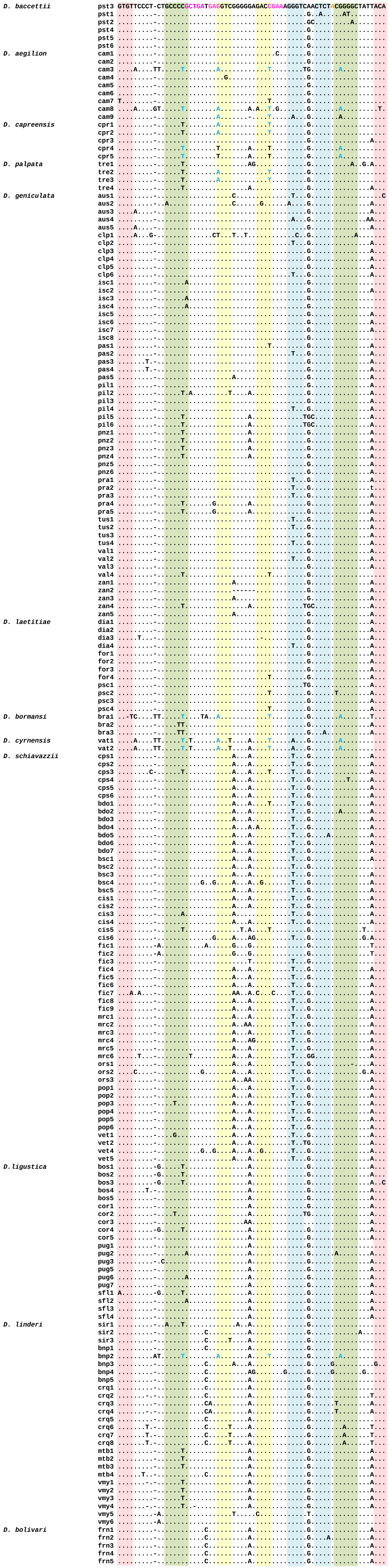

D. baccettii
pst3 GTGTTCCCT-CTGCCCCGCTGATGAGGTCGGGGGAGACCGAAAGGGTCAACTCTACGGGGCTATTACA
D. aegilion
D. capreensis
D. palpata
D. geniculata
D. laetitiae
D. bormansi
D. cyrnensis
D. schiavazzii
D.ligustica
D. linderi
D. bolivari
